# Low bone mineral density of the spine in adolescents with cerebral palsy relates to reduced correction of scoliosis after surgery

**DOI:** 10.1038/s41598-025-17269-7

**Published:** 2025-08-27

**Authors:** Konstantinos Tsaknakis, Charlotte Scheulen, Katja A. Lüders, Heide Siggelkow, Heiko M. Lorenz, Lena Braunschweig, Anna K. Hell

**Affiliations:** 1 Paediatric Orthopaedics, Department of Trauma, Orthopaedic and Plastic Surgery, University Medical Center Goettingen, Georg-August-University, 37075 Goettingen, Germany; 2 MVZ Endokrinologikum Goettingen, Goettingen, Germany

**Keywords:** Cerebral palsy, Scoliosis, Osteoporosis, Bone mineral density, Pathological Fracture, Adolescents, Orthopaedics, Paediatrics

## Abstract

Adolescents with cerebral palsy (CP) often require scoliosis surgery. Low bone mass may counteract benefits of surgical treatment. This study compares volumetric bone mineral density (vBMD) in adolescents with CP to age and sex matched healthy controls and evaluates its effect on scoliosis treatment. Computed tomogramms (CT) of 51 adolescents with CP (15.0 ± 2.6 years) were performed for scoliosis surgery and also used for vBMD calculation. Reference control vBMD values were calculated from 62 CT examinations of patients (15.1 ± 2.3 years) after trauma or conditions not related to bone mass. Z-scores were calculated based on the reference values. Correction of scoliosis in relation to vBMD was evaluated on perioperative spinal radiographs of operated adolescents with CP. Adolescents with CP had lower vBMD (123.3 ± 46.3 mg/cm^3^) than healthy controls (166.9 ± 31.4 mg/cm^3^). The lowest vBMD (97.3 ± 49.8 mg/cm^3^) had patients with CP and pathological fractures (*n* = 8). Male CP Z-scores (− 2.2 ± 1.6, *n* = 22) (16.2 ± 2.5 years) were significantly lower than female CP Z-scores (− 1.0 ± 1.3, *n* = 29) (14.1 ± 2.3 years). Higher vBMD (179.2 ± 45.4 mg/cm^3^, *n* = 41) correlated to scoliosis correction > 50% (average 67.0 ± 12%), while lower vBMD (134.9 ± 30.9 mg/cm^3^, *n* = 7) related to correction ≤ 50% (average 36.8 ± 14%). Non-ambulant adolescents with CP have lower vBMD values compared to a healthy population, which negatively affects surgical correction of scoliosis.

Level of evidence/clinical relevance: Therapeutic Level III.

## Introduction

Adolescent patients with CP and advanced restriction or complete loss of ambulation often develop severe secondary scoliosis, which requires surgical correction to relieve pain and to avoid loss of sitting ability, reduction of vital lung capacity and impaired bowel movement. Such patients are mostly or completely wheelchair-bound, with ambulation and posture control corresponding to levels III, IV and V of Gross Motor Function Classification System (GMFCS) as described by Palisano et al. in 1997^1^.

**Fig. 1:**
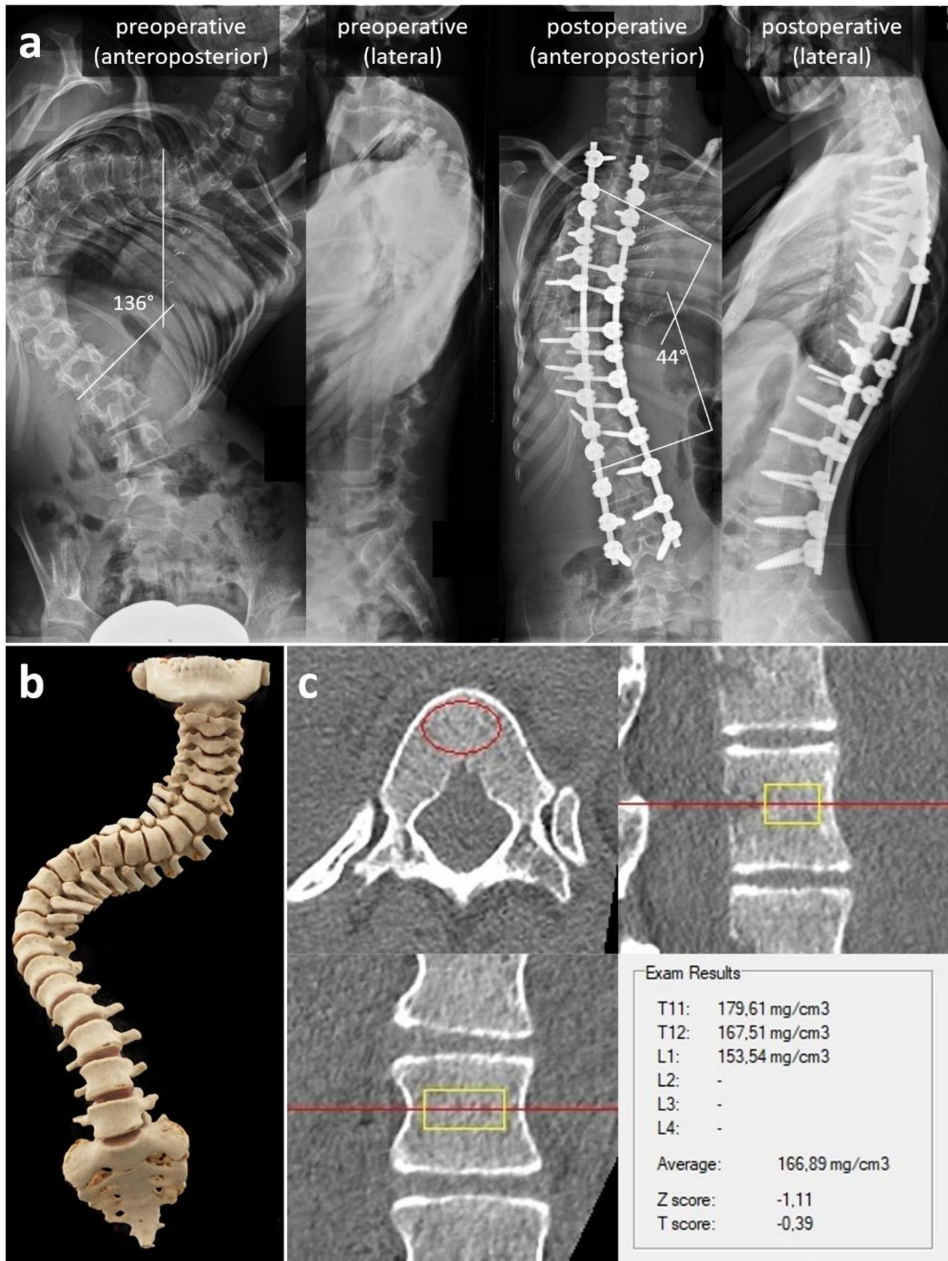
(**a**) Preoperative and postoperative anteroposterior and lateral sitting radiographs of a 13 years female patient with cerebral palsy, with correction of scoliosis from an angle of 136° to 44° after extensive spinal instrumentation and fusion from T (thoracic) 2 to L (lumbar) 5 vertebra, which equals to 68% scoliosis correction. (**b**) The spine model reconstructed from computed tomography data shows severity of spinal misalignment in all three dimensions. Therefore, in order to extract volumetric bone mineral density values (**c**), accurate alignment on all three anatomical planes is required for each vertebral body to be measured. Red line in sagittal and coronal plane represents the level of the cross-section seen in axial plane. The red circle in axial plane defines the circumference and yellow rectangles in sagittal and coronal planes define height, depth and width of the volume of the range of interest, in which bone mineral density is measured. This volume must be centered on the vertebral body and may only contain cancellous bone, leaving out neurovascular structures and cortical bone.

The gold standard treatment for progressive spinal decompensation in adolescents with CP currently consists of an extensive spinal instrumentation and fusion (Fig. 1). Because of advanced scoliosis, vertebral rotation and hypoplastic pedicle structures computed tomography (CT) may be performed in sedation for preoperative evaluation, as well as for intraoperative navigational assistance. Precalibration of CT scans allows for accurate calculation of volumetric Bone Mineral Density (vBMD) in adolescents with CP despite severe spinal asymmetry, positioning problems and reduced compliance, which otherwise pose a limitation for areal BMD (aBMD) measurements with conventional Dual Energy X-ray Absorptiometry (DXA)^2^.

The purpose of this study is to provide data on vBMD of adolescent patients with CP and scoliosis compared to a healthy age-matched population, through quantitative computer tomography (QCT) based measurements in the cancellous bone of thoracic and lumbar vertebral bodies. Influencing factors as well as the effectiveness of posterior spinal instrumentation and fusion are evaluated in relation to vBMD.

## Methods

All cases of adolescents with CP were enrolled in the period between 2017 and 2023, who were scheduled for surgical correction of scoliosis and therefore routinely received a CT examination of the spine for preoperative planning and intraoperative navigated pedicle screw placement. Inclusion criteria for the study were diagnosis of CP as defined by Bax et al.^3^, advanced restriction of ambulation with a GMFCS level of III, IV or V^1^, progressive scoliosis requiring posterior spinal instrumentation and fusion to alleviate pain and/or preserve wheelchair ambulation and/or improve lung capacity. Furthermore all included patients had received a preoperative spinal CT, which was precalibrated for calculation of vBMD. None of the patients had prior surgical correction of scoliosis.

Datasets of CT scans of healthy controls were evaluated for comparison and interpretation of vBMD and Z-scores calculation. Therefore, an age- and sex matched cohort of controls was analysed with absence of disease with possible effect on bone anatomy or quality. Patients with adolescent idiopathic scoliosis (AIS) did not deem as a control group, since AIS has a different pathomechanism from secondary scoliosis and is known to innately have lower BMD^4,5^. Trauma CT scans were used for extracting reference values. Since an injured person with lower BMD has a higher probability for a fracture and complaints that may lead more often to a CT examination, this could prove a bias for the reference values. For this reason, trauma related datasets derived only from polytrauma-CT scans performed as standard protocols without exception after high velocity/energy trauma in high risk, emergency situations, which rules out behavioral bias.

CT scanners (Somatom Definition AS, Siemens, Erlangen, Germany) were precalibrated regularly with a patented phantom required by software QCTpro^®^ version 6.1 (Mindways Software Inc., Austin, TX, USA), in order to interpret CT datasets into mg/cm^3^ calcium phosphate.

All CT scans of patients with CP were evaluated by two independent and experienced examiners in order to calculate an average vBMD and compensate for inter-observer variability. Each examiner would define a range of interest (ROI) strictly in cancellous bone of all levels from the first thoracic to the fifth lumbar vertebra, after having performed an alignment in all anatomical planes for each thoracic and lumbar vertebra individually (Fig. 1). Alignment in all anatomical planes means that the examiners had to bring the default planes of the CT examination, which are defined by the position of the CT scanner table, to exactly overlap with the anatomical axial, coronal and sagittal plane of the vertebra as shown in Fig. 1. This was done during the step of multiplanar reconstruction of the CT data in software QCTpro^®^. Since scoliotic vertebrae have different rotation and inclination, this adjustment had to be painstakingly performed for each vertebra from T1 to L5. In order to estimate intra-observer variability a sample of CT datasets was reevaluated at least two months apart by each examiner.

In the reference group, native and contrast agent enhanced CT scans (Imeron^®^ 350 mg/ml, Bracco Imaging Deutschland GmbH, Konstanz, Germany or Ultravist^®^ 370 mg/ml Bayer Vital GmbH, Germany, Leverkusen) were processed. Control group vBMD was calculated by one of the two previous examiners, since normal spine anatomy made orientation and ROI definitions less challenging.

Contrast agent enhanced CT scans led to artificially higher vBMD (BMD_MDQCT_) measurements, which were converted using the according to equation of Bauer et al.^6^:

$${\text{BMD}}_{{{\text{QCT}}}} = 0.{\text{96}} \times {\text{BMD}}_{{{\text{MDQCT}}}} - {\text{2}}0.{\text{9 mg/ml}}$$

Since males and females within our CP cohort were not perfectly age-matched, we calculated the Z-score to compensate for the age difference when comparing levels of bone mass between the two sexes. Z-score allows an age and sex matched comparison of results, based on age and sex matched average vBMD control values and their standard deviation (SD). Calculations were performed for each measured vertebral vBMD value.

Z-score expresses how many control group SDs (SD_CTRL_) is a patient’s vBMD (BMD_CP_) above or below the average value of the matched control group (BMD_meanCTRL_)^7^:

$${\text{Z - score}} = \left( {{\text{BMD}}_{{{\text{CP}}}} - {\text{BMD}}_{{{\text{meanCTRL}}}} } \right)/{\text{SD}}_{{{\text{CTRL}}}}$$

QCTpro^®^ according to user’s manual information provides Z-scores from reference vBMD values of University of California, San Francisco (UCSF) dating back to 1985–1988^8–12^ and only for lumbar vertebrae. Especially for children the reference data derive only from two of these studies in 1986 and 1988^11,12^. The sample in the first study were 101 children. For the second study there was no data available. For this reasons vBMD data of a local and concurrent control group including thoracic vertebrae values were used for calculations, instead of the original reference values. A compensation for growth with height-adjusted Z-scores (HAZ)^13^ in QCT-based vBMD was not required, since all three dimensions are already taken into account.

Standardized pre- and postoperative anteroposterior and lateral sitting radiographs of the spine were performed and scoliosis angles of the main curve were measured^14^ in order to determine a possible influence of vBMD on surgical outcome (Fig. 1). The examiner evaluating the radiographs was one of the senior surgeons involved in the surgical treatment of the patients with CP.

Surgical correction was performed by three experienced pediatric spine surgeons applying the same technique. Posterior spinal instrumentation and fusion was usually performed from the second thoracic to the fifth lumbar vertebra with bilateral placement of polyaxial and/or uniplanar pedicle screws in almost all levels (Fig. 1). Screw placement was performed under navigation guidance for which the preoperative CT scans were used. Correction of scoliosis was made with 5.5 mm diameter Titanium alloy rods, which allow for more flexibility and reduce the risk of screw loosening because of reduced bone mass. Pelvic fixation was not performed in any of the involved cases because of low body mass index (BMI), small pelvic bone and the high risk for decubitus and soft tissue perforation.

Patients with a correction of at least 50% of the main spinal curve were compared to those with correction of 50% or less. Literature does not define an expected minimum of surgical scoliosis correction. However, it does offer evidence that an effective brace treatment in AIS should achieve a 50% initial angle correction^15,16^. Therefore, one would expect surgical scoliosis correction with posterior spinal instrumentation and fusion should achieve main curve reduction above 50%, since the spine is being directly manipulated. Angle measurements were performed using Centricity Enterprise Web Version 3.0 (GE Healthcare Medical Systems, Chicago, United States, 2006).

In order to examine further possible vBMD predictors, medical records were reviewed and families were contacted for additional information or interviewed upon admission. Consent was implied through acceptance of interview participation. Besides age, sex, GMFCS level and BMI data also included additional parameters as shown in Table 1. Bone influencing blood parameters at time of the CT examination including creatinine, alkaline phosphatase, calcium and thyroid stimulating hormone (TSH) were analyzed.

BMI was calculated through division of patient´s measured body weight in kilograms with squared height in meters. Height was calculated as total length of straight lines connecting top of the head to trochanter major, to lateral femur condyle and to calcaneus, in order to adjust for joint contracture. Evaluation of BMI was based on reference tables for adolescents and children as published by Coners et al.^17^.

After acquisition of all data, CP patients were divided into subgroups for each parameter (e.g. low BMI vs. normal BMI; creatinine below vs. above a threshold), while checking whether the other parameters were equally distributed among the two groups. Then, the vBMD values between these groups were compared.

Statistical analysis was performed with the software GraphPad Prism^®^ and Excel^®^ (Microsoft Corporations, Redmond, USA) using an unpaired t-Test to compare vBMD and Z-Score results. vBMD in the text is presented as mean ± standard deviation and it represents the value calculated form all vertebrae from T1 to L5. Statistical significance was determined as *p* ≤ 0.05(*), *p* ≤ 0.01(**) and *p* ≤ 0.001 (***).

 All procedures performed in this studies involving human participants were in accordance with the ethical standards of the institutional and/or national research committee and with the declaration of Helsinki and its later amendments or comparable ethical standards. The Institutional Ethics Committee of University Medical Center Göttingen approved the study (reference number 20/4/21). The need for written informed consent was waived by Institutional Ethics Committee of University Medical Center Göttingen.

## Results

A total of 51 adolescents with CP and scoliosis were included in this study, with an average age at time of CT examination of 15.0 ± 2.6 years (males 16.2 ± 2.5 years; females 14.1 ± 2.3 years) (Table 1) and with an average BMI of 16.4 ± 3.9 kg/m^2^ (males 15.7 ± 3.1; females 16.7 ± 4.1). All 51 cases were included in QCT evaluation of vBMD. Reported pathological fractures in eight cases were located in the extremities only, so that all vertebrae could be included in vBMD measurements. Another eight initially planned participants had to be excluded, either because parents decided against a surgical treatment of scoliosis and thus not requiring a CT scan (*n* = 5), or because of technical inconsistencies of CT calibration for vBMD measurements (*n* = 3). Sampled re-evaluation of vBMD measurements on 11 CT datasets showed an intra-observer variability of 4.8% and 5.3% respectively and an inter-observer variability of 5.0%.

During the study period, surgical scoliosis correction was performed in 94% (*n* = 48) of CP patients. Preoperative radiographs were taken 42 ± 29 days prior to intervention. Postoperative corrections were measured on radiographs immediately and on average 11 ± 8 days after surgery. Follow-up time was on average 13 ± 9 months. Complications arose in two cases. In the first case a decubitus developed after nine months because of dislocation of the left pedicle screw of the fifth lumbar vertebra in combination with a severely low BMI. The screw was surgically removed and the rod was shortened without further problems. In the other case a secondary, subacute infection of the implants occurred one year after surgery and the implants had to be removed and the patient received antibiotic treatment.

For reference vBMD values and Z-scores calculation 62 CT scans of healthy participants aged 15.1 ± 2.3 years (males 15.7 ± 2.3 years; females 14.5 ± 2.2 years) were chosen for evaluation, so that would be age-matched to the patients with CP as shown in Table 1. Of these CT scans 28 were native and 34 were contrast agent enhanced examinations. Datasets included 26 polytrauma CTs, 23 CTs of thorax/abdomen/pelvis, 11 spine CTs, 1 biopsy CT and 1 heart CT. Bone related findings were fractures of T5 to T7 in one case and two cases of spondylolisthesis in segment L5-S1. The involved vertebrae were not analyzed and excluded. Bone irrelevant pathological findings were urinary tract infections (*n* = 8), primary lung embolism (*n* = 3), pneumothorax (*n* = 1) and atrial septum defect (*n* = 1). The number of CT scans in the control group had to be larger to reach an adequate number of vertebrae evaluated on vBMD, than in the group with patients with CP, because in many cases only part of the spine was scanned, if for example a CT examination only of the abdomen or the chest was available.

**Table 1:** Table shows demographic data for patients with cerebral palsy and of the healthy control group, as well as parameters taken into consideration as possible predictors for low volumetric bone mineral density, with resulting patient distribution in absolute numbers (n) and percentages.

Analysis Criteria		Cerebral palsy (*n* = 51)	Control (*n* = 62)
Sex	Male	*n* = 22	*n* = 31
	Female	*n* = 29	*n* = 31
Age	All	15.0 ± 2.6 year	15.1 ± 2.3 year
	Male	16.2 ± 2.5 year	15.7 ± 2.3 year
	Female	14.1 ± 2.3 year	14.5 ± 2.2 year
Gross Motor Function Classification System (GMFCS)		III *n* = 2 (4%)	–
		IV *n* = 6 (12%)	
		V *n* = 43 (84%)	
Scoliosis angle		95° ± 24° preoperative	–
		36° ± 18° postoperative	
		Average correction 62.6%	
Pelvic obliquity		27° ± 20° preoperative 12° ± 12° postoperative	
Pathological fractures		Yes *n* = 8 (16%)	–
		No *n* = 43 (84%)	
Epilepsy		Yes *n* = 40 (78%)	–
		No *n* = 11 (22%)	
Antiepileptic therapy		Yes *n* = 38 (75%)	–
		No *n* = 13 (25%)	
Baclofen therapy		Oral *n* = 7 (14%)	–
		Intrathecal *n* = 11 (21%)	
		No *n* = 33 (65%)	
Vit. D min. 1000IU ≥ 1 year		Yes *n* = 36 (71%)	–
		No *n* = 15 (29%)	
Gastrostomy		Yes *n* = 30 (59%)	–
		No *n* = 21 (41%)	
Non-invasive ventilation		Yes *n* = 2 (4%)	–
		No *n* = 49 (96%)	

**Fig. 2:**
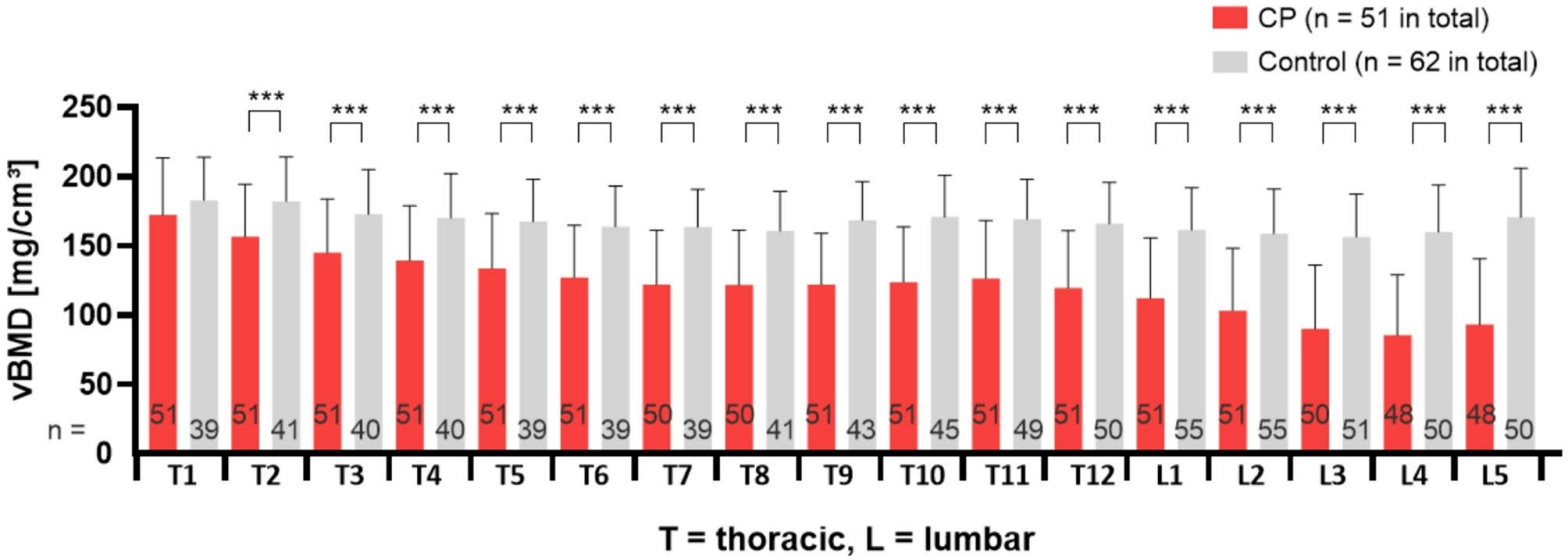
Comparison of volumetric bone mineral density values of each thoracic (T1–T12) and lumbar (L1–L5) vertebra of group with CP (red) to age- and sex-matched controls (grey). Differing numbers in grey columns represent available vBMD control values for each level, since they are derived from various types of CT scans. Differing numbers in red columns result from excluded vertebrae because of artefacts. *** *p* ≤ 0.001.

The average spinal vBMD of the group with CP (123.3 ± 46.3 mg/cm^3^, range 7–271 mg/cm^3^) was significantly lower in comparison to healthy controls (166.9 ± 31.4 mg/cm^3^, range 88–293 mg/cm^3^), corresponding to an average Z-score of − 1.5 ± 1.5. Except for thoracic vertebra 1, all other thoracic and lumbar vertebrae showed significantly lower vBMD values in patients with CP in comparison to healthy controls. The magnitude of the difference between CP patients and controls increased in the cranial to caudal direction from a 16% to over 80% higher vBMD in controls compared to CP patients (with the highest difference in L4 with 87% and L5 with 83% higher vBMD in controls) (Fig. 2).

### vBMD and Z-scores in male and female adolescents with CP

**Fig. 3:**
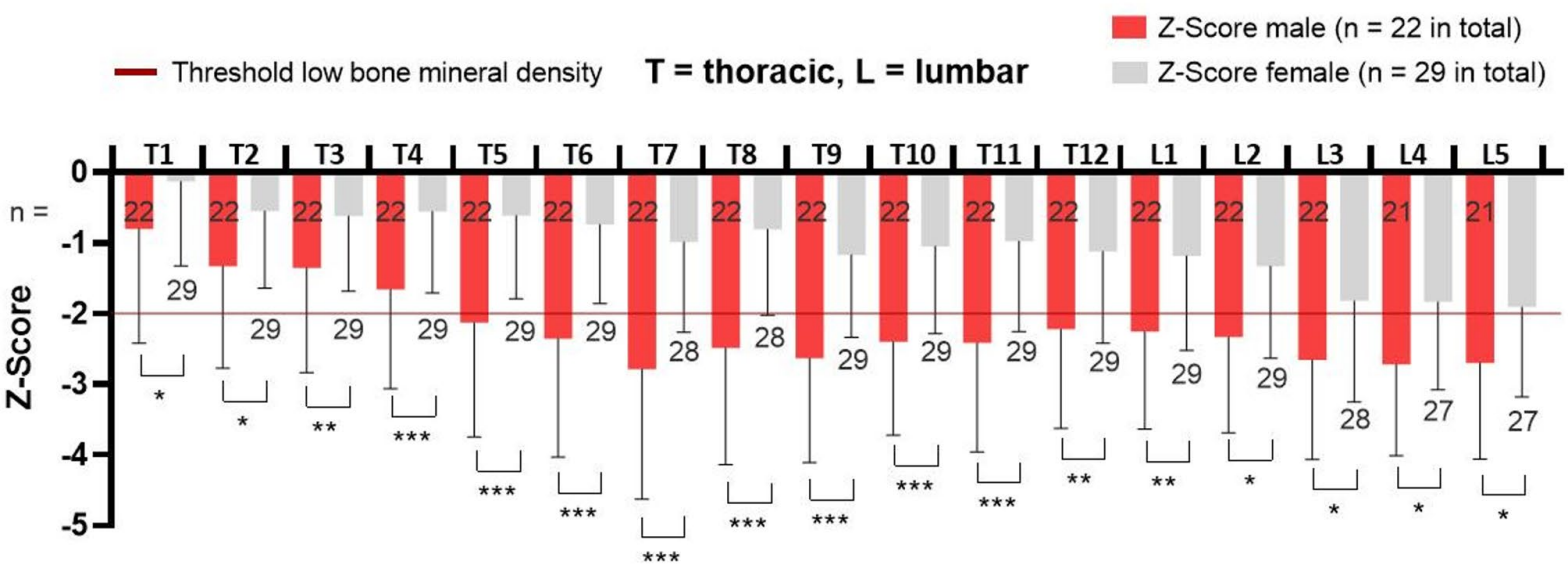
Z-scores in patients with cerebral palsy. Female average Z-score values for each vertebra (grey bars) remain above threshold of − 2 SDs for low BMD (red line), while in male patients (red bars) Z-scores mostly lay below it. Differing numbers in some columns result from excluded vertebrae because of artefacts. *** *p* ≤ 0.001, ** *p* ≤ 0.01, * *p* ≤ 0.05.

Further discrimination according to sex showed that male patients (*n* = 22) had lower vBMD (112.3 ± 43.8 mg/cm^3^) in comparison to male controls (167.1 ± 27.4 mg/cm^3^, *n* = 31). Corresponding average Z-score for all vertebrae was − 2.2 ± 1.6. However, difference was not so pronounced when comparing 29 female patients with CP with overall vBMD of 131.8 ± 46.4 mg/cm^3^ to their 31 healthy counterparts with a vBMD of 166.9 ± 35.2 mg/cm^3^, which corresponded to an average Z-score of -1.0 ± 1.3. In both groups, Z-scores were more favorable in the cranial compared to the more caudal vertebrae. Still, female patients demonstrated more favorable results in comparison to males at each level. Male patients Z-scores laid below threshold for low BMD in children^2^ as shown in Fig. 3. The mean estimates for females did not reach the Low BMD threshold at any level.

### Correlation of pathological fractures and vBMD

**Fig. 4:**
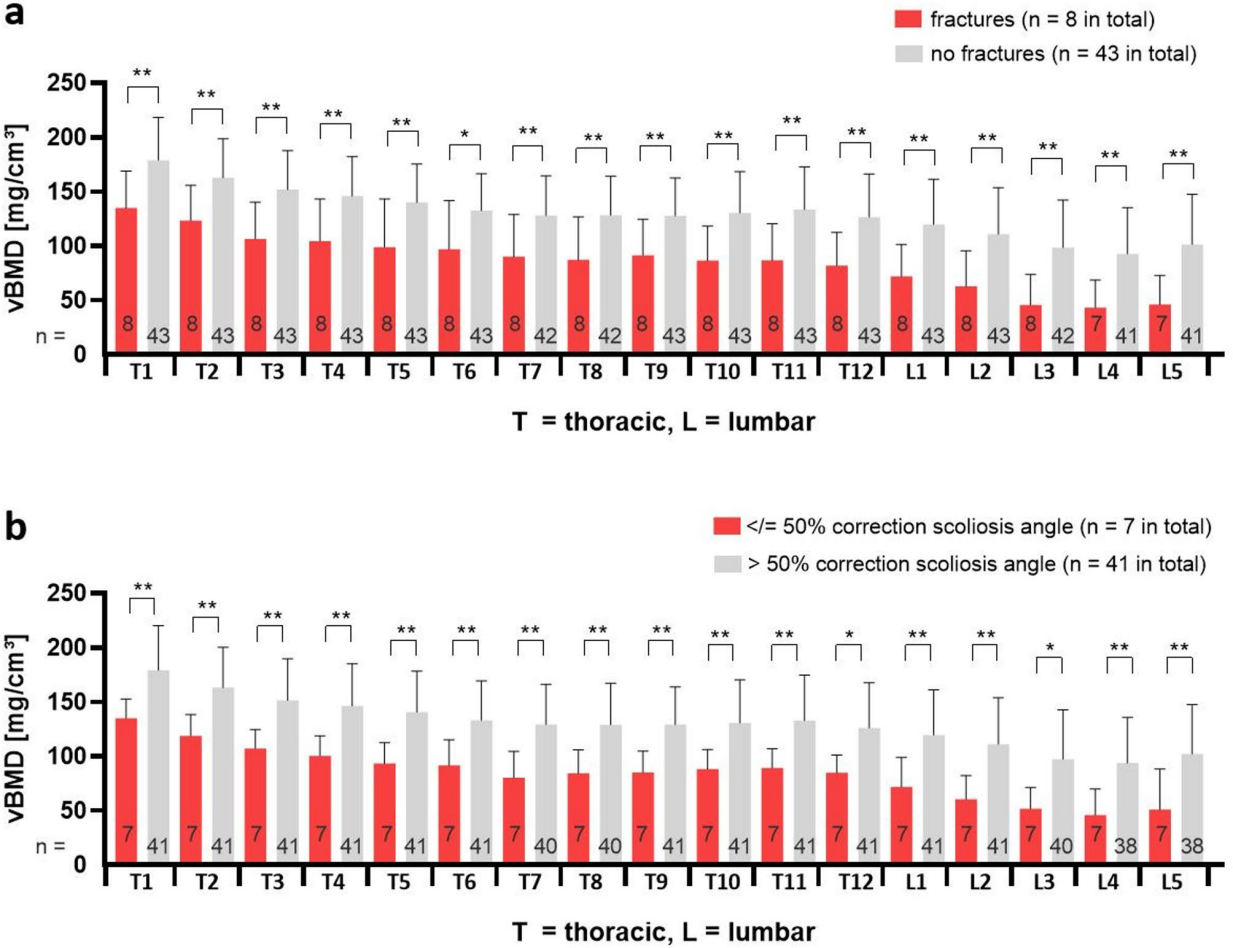
(**a**) vBMD values of each thoracic (T1–T12) and lumbar (L1–L5) vertebra in patients with cerebral palsy and pathological fractures (*n* = 8) are shown in red and those of patients without fractures (*n* = 43) in grey. All vertebrae in patients with previous fractures have significantly lower vBMD. (**b**) Patients with surgical correction outcome of ≤ 50% of preoperative scoliosis angle (red) had significantly lower vBMD for each thoracic and lumbar vertebra in comparison to patients with scoliosis correction > 50% (grey). Differing numbers in some columns result from excluded vertebrae because of artefacts. T = thoracic. L = lumbar. ** *p* ≤ 0.01, * *p* ≤ 0.05.

Adolescents with CP and a history of pathological fractures (*n* = 8) had significantly lower spinal vBMD values (97.3 ± 49.8 mg/cm^3^, range 6.6-245.6 mg/cm^3^) in comparison to patients without pathological fractures (*n* = 43) (130.2 ± 44.0 mg/cm^3^, range 11.9-270.9 mg/cm^3^) (Fig. 4). The average Z-score in patients with fractures was − 2.7 ± 1.5.

### Influence of vBMD on outcome of surgical scoliosis correction

A total of 48 adolescents with CP underwent surgical scoliosis correction by means of posterior spinal instrumentation and fusion. Initial angle of main curve was reduced on average 62.6%, from 95° ± 24° to 36° ± 18° postoperatively. 41 adolescents with average preoperative scoliosis of 93° ± 22° had a correction above 50%, to 31° ± 14° (average 67.0%), whereas seven patients had a correction of 50% or less, from 105° ± 34° to 64° ± 17° (average 36.8%). The latter group had significantly lower overall vBMD values (134.9 ± 30.9 mg/cm^3^, range 11.9–160.8 mg/cm^3^) in comparison to vBMD of the first group (179.2 ± 45.4 mg/cm^3^, range 10.7–270.9 mg/cm^3^) (Fig. 4). Further analysis was required in order to attribute lower correction potential to lower vBMD. Simple linear regression analysis showed no correlation of the preoperative scoliosis angle to the achieved percentage correction (R^2^ = 0.01). Furthermore, both correction groups (> 50% versus < 50%) showed otherwise no statistically significant differences in any of the analysis criteria (Table 1). Ratio of female to male patients with CP in both groups was 1.3. Average age was 15.0 ± 2.6 year and 15.2 ± 3.0 year (*p* > 0.05) respectively, creatinine average values were 0.44 ± 0.15 mg/cm^3^ and 0.38 ± 0.12 mg/cm^3^ (*p* > 0.05) and average BMI was 16.6 ± 4.0 kg/m^2^ and 15.2 ± 4.0 kg/m^2^ (*p* > 0.05). Finally, the group with lower correction consisted only of patients with GMFCS V. Therefore, a subgroup analysis of GMFCS V patients with high scoliosis correction (*n* = 33) in comparison to poor correction (*n* = 7) was performed, which showed that the achieved postoperative scoliosis angle of 31 ± 13° with an average correction of 66.4% was not influenced by the GMFCS level.

### Correlation of serum creatinine level to vBMD

**Fig. 5:**
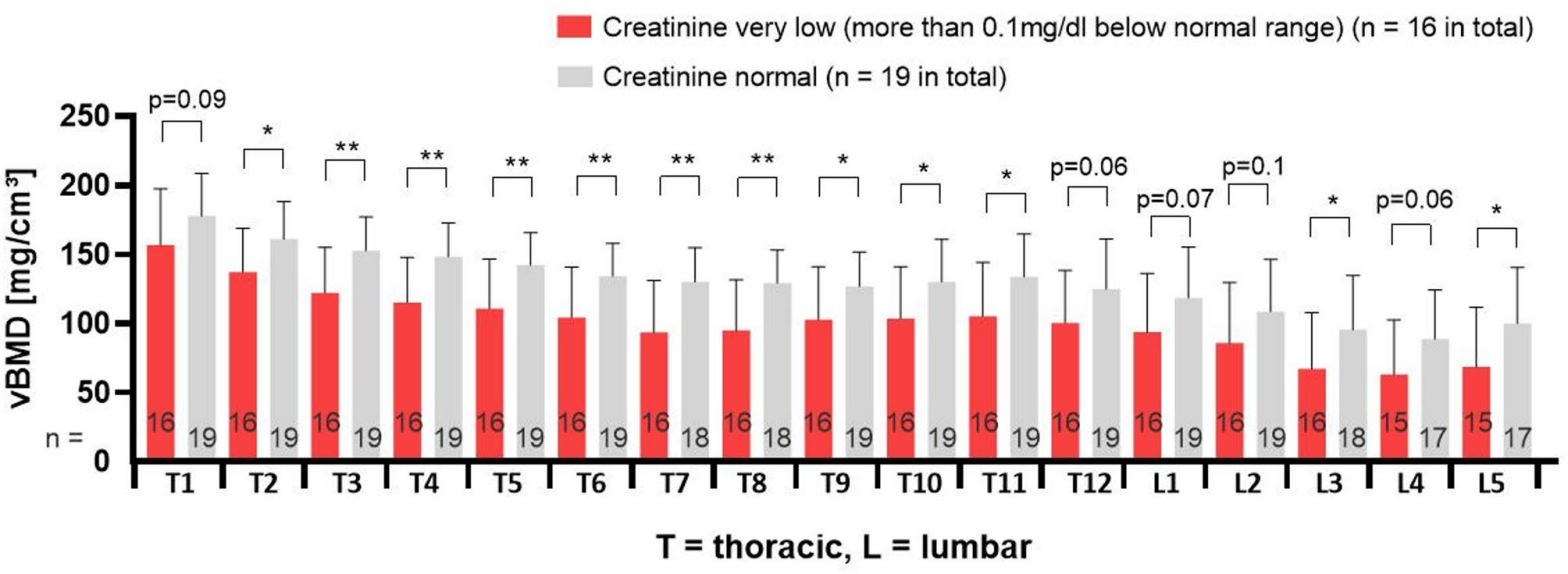
Patients with cerebral palsy and very low creatinine values also had significantly lower vBMD in most examined vertebrae (red) than adolescents with creatinine in normal range (grey). Differing numbers in some columns result from excluded vertebrae because of artefacts. T = thoracic. L = lumbar. ** *p* ≤ 0.01, * *p* ≤ 0.05.

Creatinine was the only value which correlated significantly to vBMD. Analysis of serum creatinine concentration in relation to vBMD showed that 16 patients with levels of at least 0.1 mg/dl lower than age and sex specific minimum normal value also had significantly lower vBMD ((101.9 ± 35.1 mg/cm^3^, range 5.7-167.3 mg/cm^3^), when compared to patients with normal creatinine values (vBMD = 130.5 ± 25.3 mg/cm^3^, range 91.3–187.9 mg/cm^3^, *n* = 19). Difference was statistically significant for most of evaluated vertebrae, as shown in Fig. 5.

Analysis of the remaining criteria as listed in Table 1 only showed significant results in patients with Levetiracetam treatment, who had partially lower vBMD in a few thoracic vertebrae than patients without antiepileptic therapy (significant differences T4–T7; *p* < 0.05; 33–36% lower vBMD in CP patients compared to controls). Comparing separately patient groups of GMFCS level V, low body mass index (BMI), epilepsy, baclofen treatment, gastrostomy, non-invasive ventilation and no vitamin D substitution, against adolescents of GMFCS level III/IV, normal BMI, without epilepsy, without gastrostomy, without non-invasive ventilation and with vitamin D supplementation respectively showed no significant reduction of vBMD values.

## Discussion

While the problem of reduced bone quality and loss of bone mass is widely known for older people, this problem has only recently come into focus for children with neuromuscular diseases^18–20^. Reduced bone mineral density (BMD) can be assumed in patients with CP of advanced GFMCS levels. aBMD can be measured using the traditional DXA method, which can be challenging in severely affected patients because of advanced spinal asymmetry, contractures and reduced compliance in cases of mental disability. For example, studies on the effect of vertebral rotation on aBMD in adults with lumbar scoliosis have shown deviations between adjacent vertebrae of at least one SD and deem results unreliable^21,22^. In fact, the International Society for Clinical Densitometry (ISCD) guidelines discourage DXA examinations, when appropriate and safe positioning of the child cannot be assured^2^. Most literature currently focus on two dimensional aBMD examinations of the lumbar region to evaluate BMD in patients with CP^23,24^. In this study three dimensional QCT measurements were performed of all thoracic and lumbar vertebrae of adolescents with CP and scoliosis, which allowed us to overcome the afore mentioned technical problems.

Analyzing QCT, children with CP had significantly lower vBMD values of the thoracic and lumbar spine compared to healthy controls and an accordingly low average Z-score of -1.9 ± 1.4 of the lumbar area L1 to L4. These results are similar to findings of 43 younger children with CP (average age 8 years), who had a lumbar Z-score of − 1.74 ± 0.15^25^. Adolescents with CP and medical history of previous pathological long bone fractures (*n* = 8) displayed the lowest overall Z-score (− 2.7 ± 1.5). Henderson et al. reported 2010 in a multicenter study with 179 DXA examinations of children with CP and previous fractures a lumbar Z-score of − 2.5 ± 1.7^26^, which is in line with our findings.

Age normalized analysis showed more than one standard deviation reduction of Z-score in male patients (− 2.2 ± 1.6) compared to female patients (− 1.0 ± 1.3). In this investigation, girls were on average two years younger than boys. Tanner pubertal development stadium^27^ data were not available for consideration in this study, nor did radiographs allow collection of sufficient growth information based on ossification of the iliac crest^28^. Examining available literature from this aspect, one would expect male patients in the present study, being on average two years older than female patients, to have higher vBMD, since they tend to achieve peak bone mass in puberty earlier than girls^29^. However, in patients with CP and severe disability undernourishment must be taken into consideration, even though BMI and gastrostomy had no significant influence on vBMD in this evaluation. According to longitudinal studies malnourished CP patients did not demonstrate as steep an increase in BMD as their healthy peers during puberty, which resulted in constantly decreasing Z-scores^26,30,31^. These findings are also in line with a correlation of low serum creatinine concentration to low vBMD values. Studies have shown that low serum creatinine levels are related to low BMD, since both are the result of reduced lean muscle mass^32^, which can be commonly seen in CP patients with undernutrition.

Using titanium rods for posterior surgical correction in idiopathic scoliosis, an average improvement of 67–76% can be expected^33,34^. However, to our knowledge, there is no literature focusing on the correction potential of neuromuscular scoliosis and BMD. Dividing CP patients into groups with good correction of the main scoliotic curve (above 50%) versus poor surgical results (equal or less than 50%), an average correction of 67.0% was found in the first group, which is in accordance with above reported results for idiopathic scoliosis. The latter population had an average correction of 36.8%, almost half of the correction of the first group. As demonstrated above, both groups only differed significantly concerning vBMD values (Fig. 4), with poor bone mass being present in the second group. Taking into account that other compared predictors were not different between the two groups, these findings support our empirical observation, that low bone mass had a negative effect on the surgical correction of scoliosis.

Limitations of the present study were the relatively small number of patients with CP and unequal distribution of participants for analysis of cofounding factors, which may affect statistical results. A further limitation was also the small sample size of the control group (*n* = 62), which may affect the accuracy of the average reference values. However, the pediatric sample size of the software QCTpro^®^ was only marginally lager (*n* = 101)^11,12^. It should also be acknowledged that our study includes many comparisons with a modest sample size and without adjusting Alpha for the multiple comparisons. A further limitation was that male and female patients with CP were not perfectly age matched, so that a comparison could be made only through their respective average Z-scores. Also, there is always the risk that indications leading to CT scans in the control group may have been related to BMD which could affect the generalizability of the results.

## Conclusion

To our knowledge for the first time, this investigation provides vBMD data of the whole thoracic and lumbar spine in adolescents with CP and scoliosis. These results support the observations that CP patients’ vBMD values do not achieve the expected levels during pubertal growth, probably because of malnutrition and disability, among other factors. Patients with pathological fractures also have very low vBMD values. Furthermore, low vBMD negatively affects the outcome of surgical scoliosis correction in CP adolescents. Adjusting medical therapy of these patients to better support bone quality in the future could possibly counteract these negative effects.

## Data Availability

The data that support the findings of this study are available from the corresponding author upon reasonable request.

## Abbreviations

aBMD: Areal bone mineral density
AIS: Adolescent idiopathic scoliosis
BMD: Bone mineral density
BMI: Body mass index
CP: Cerebral palsy
CT: Computed tomography
DMD: Duchenne muscular dystrophy
DXA: Dual energy X-ray absorptiometry
GMFCS: Gross motor function classification system
HAZ: Height adjusted Z-scores
ISCD: International society for clinical densitometry
MRI: Magnetic resonance imaging
QCT: Quantitative computed tomography
ROI: Range of interest
SD: Standard deviation
TSH: Thyroid stimulating hormone
UCSF: University of California, San Francisco
vBMD: Volumetric bone mineral density
